# Pep19: A Novel Approach for Reducing Visceral Fat and Improving Sleep Quality in Obese Adults—Results From an Early‐Stage Clinical Trial

**DOI:** 10.1002/dmrr.70056

**Published:** 2025-06-01

**Authors:** Andrea S. Heimann, Prachi Singh, Emer S. Ferro, Frank Greenway, Arnon Krongrad

**Affiliations:** ^1^ Proteimax Biotechnology Israel Caesarea Israel; ^2^ Pennington Biomedical Research Center Baton Rouge Louisiana USA; ^3^ Pharmacology Department Biomedical Science Institute University of Sao Paulo Sao Paulo Brazil

**Keywords:** body composition, intracellular peptide, obesity, sleep quality, visceral fat

## Abstract

**Background:**

Conformational‐sensitive antibodies were used to identify the orally active peptide DIIADDEPLT (Pep19) as an inverse agonist of cannabinoid type 1 receptor. Pep19 safely improved metabolic parameters in murine models of diet‐induced obesity, and in healthy dogs.

**Objectives:**

To evaluate Pep19's impact on quality of life and body composition in obese adults, hypothesising that the metabolic effects of Pep19 observed in animal models could translate to humans.

**Methods:**

Subjects, males (*n* = 12) and females (*n* = 12), from 46 to 59 years old, weighing 91–106 kg, body mass index between 30 and 35 kg/m^2^, were evaluated over 60 days in a placebo‐controlled, triple‐blinded clinical trial; participants received either a placebo, 2 or 5 mg Pep19 capsules once daily at bedtime. The primary endpoint was a broad measure of quality of life assessed using validated questionnaires. The key secondary endpoints included weight loss, reduction in visceral fat (measured by dual‐energy X‐ray absorptiometry), and changes in waist, hip, and chest measurements.

**Results:**

Pep19 was well tolerated with no reported adverse effects. Remarkable reductions in visceral fat were observed in the 5 mg Pep19 group, with a 17 ± 4.7% loss (*p* < 0.05), without any change in lean mass. Additionally, sleep quality improved significantly by 35 ± 10% in the 2 mg Pep19 group and 25 ± 16% in the 5 mg Pep19 group (*p* < 0.05). In the 5 mg Pep19 group, significant reductions in body weight and waist circumferences were also observed (*p* < 0.05).

**Conclusion:**

Despite the limitations related to the use of convenience sampling, a small sample size, and a short intervention duration, which may restrict generalisation and health claims, Pep19 demonstrates exceptional innovative potential as a novel approach to reduce visceral fat and improve sleep quality.

## 1 Introduction

Peptides serve as natural ligands for numerous cell surface and intracellular targets, playing critical roles in physiology, diagnosis and therapeutics [1, 2, 3, 4, 5]. Intracellular peptides are natural bioactive compounds initially formed inside the cells from proteasomal protein degradation [6, 7, 8, 9, 10, 11, 12, 13]. Several lines of evidence suggest that intracellular peptides have both physiological and pharmacological properties [6, 13, 14, 15, 16, 17, 18]. The cyclophilin A‐derived peptide DITADDEPLT was identified in mouse abdominal fat after a high fat diet challenge [19]. Using conformational sensitive antibodies [20, 21], the DITADDEPLT peptide was characterised as having weak inverse agonist activity at the cannabinoid type 1 receptor (CB1R) [22]. A single amino acid substitution at the third position of the DITADDEPLT peptide significantly enhanced its binding affinity to CB1R as an inverse agonist, while other substitutions altered the peptide's specificity towards the cannabinoid type 2 receptor (CB2R) or converted it into an agonist of CB1R [22]. Extensive pharmacological characterisation led to the modification of the original peptide into DIIADDEPLT (Pep19), which demonstrated its ability to activate the extracellular signal‐regulated kinase 1 and 2 (pERK1/2) and protein kinase B (AKT) signalling pathways in 3T3‐L1 differentiated adipocytes [22]. Pep19 induced the expression of uncoupling protein 1 (UCP1) in both white adipose tissue and 3T3‐L1 differentiated adipocytes, with this effect being antagonised by the CBR1 receptor antagonist AM251 [22].

The accumulation of visceral fat and its relationship to obesity‐associated diseases highlights the importance of therapeutic interventions targeting visceral fat in mitigating the health risks associated with abdominal obesity [23, 24, 25]. Weight‐control interventions often demonstrate limited effectiveness in addressing obesity, primarily due to the complex physiological adaptations induced by obesity across multiple tissues, including adipose tissue, skeletal muscle, and the brain [26]. Recent studies have shown that the accumulation of visceral fat, rather than subcutaneous fat, is more strongly associated with increased cardiometabolic risk [27, 28, 29]. Another factor contributing to obesity and related disorders is sleep [30]. Experimental studies show that sleep deprivation contributes to obesity via increases in appetite, especially for foods high in calories and sugars, due to changes in hunger‐regulating hormones such as ghrelin and leptin [31]. The combination of aerobic exercise and fasting protocols may offer synergistic benefits for weight loss and sleep improvement [32, 33]. Moreover, lack of sleep can lead to decreased insulin sensitivity and increased visceral fat, creating a vicious cycle that contributes to weight gain, and additional cardiometabolic risk [34].

Oral administration of Pep19 into diet‐induced obese Wistar rats significantly reduces visceral adipose tissue, adiposity index, whole body weight, glucose, triacylglycerol, cholesterol, and blood pressure, without altering heart rate; changes in the number and size of inguinal adipocytes, with an increase in UCP1 protein, were also observed [22]. Together, these data suggested that Pep19 oral administration is turning white adipose cells into brown adipose cells [22]. Pep19 exhibits no cytotoxicity and no effects on the central nervous system, as indicated by the absence of brain c‐Fos expression induction, failure to trigger the cannabinoid tetrad, and lack of depressive‐ and anxiety‐like behaviours [22]. Pep19 has also been demonstrated to reduce weight gain, enhance insulin sensitivity, lower blood pressure, decrease liver inflammation and lipid accumulation, and promote fat browning in male Swiss mice on a high‐fat diet, all without any observable effects on central nervous system activity or behaviour [17]. In healthy adult neutered beagles (4 females and 4 males), Pep19 oral administered once daily for 28 days (5 mg/dog/day, 0.32–0.49 mg/kg/day) showed no adverse effects and all blood and urine analyses remained normal [35]. Despite no changes in diet or calorie intake, seven of the dogs lost between 0.7% and 3.8% of their body weight (*p* < 0.01). Thus, Pep19 safety and beneficial contribution to body weight reduction was further confirmed in healthy dogs [35].

Pep19 received the General Recognised as Safe/GRAS status based on scientific procedures in accordance with 21 C.F.R. § 170.30 (a) and (b) and conforms with guidance § 170.36 from the United States of America Food and Drug Administration (FDA), and became commercially available in the form of 5 mg supplement capsules (Nutroslim LLC, Doral, FL, USA). Several individuals who were taking commercially available Pep19 in the form of 5 mg supplement capsules once a day at bedtime for at least 30 days independently and spontaneously reported weight loss and reduced waist circumference along with improved sleep quality (unpublished data). Importantly, none of the subjects reported any adverse undesired effects of Pep19, which is consistent with the previously observed absence of central nervous system activation and behaviour effects on rodents and dogs [17, 22, 35]. To the best of our knowledge, this is the first demonstration of an orally active intracellular peptide exhibiting pharmacological effects that are consistent across 3T3‐L1 differentiated adipocytes, rodents, dogs, and humans.

Building on the hypothesis that Pep19's metabolic effects observed in laboratory models could translate to humans, potentially improving both the quality of life and body composition, we conducted an early stage rigorous 60‐day, triple‐blind, placebo‐controlled clinical trial with 24 obese subjects (body–mass index—BMI, between 30 and 35 kg/m^2^). Subjects were assigned to the placebo, 2 mg Pep19, or 5 mg Pep19 groups, administered orally once daily at bedtime; unlike previous studies that primarily focused on the use of small molecules or modified peptides for metabolic improvement of obese subjects, our present study has the differential of using an orally active natural peptide. Notably, 5 mg Pep19 intervention resulted in significant reductions in visceral fat, body weight and waist circumferences. Sleep quality was improved by both 2 and 5 mg doses of Pep19. These promising results suggest that Pep19 has successfully translated its remarkable effects from laboratory models to human applications, highlighting its innovative potential as a novelty to enhance overall health.

## 2 Material and Methods

### 2.1 Pep19 Synthesis and Capsules Preparation

Pep19 (DIIADDEPLT; CAS Registry Number 1536481‐46‐5: white adipose tissue activator cyclophilin A peptide—WATACAP) is a natural peptide synthetically produced using a 9‐fluorenylmethyloxycarbonyl (FMOC) solid‐phase synthesis method with active ester amino acids (Proteimax Biotechnology Israel, Caesarea, Israel) [36, 37]. Both Pep19 and placebo were formulated by Nutroslim LLC (Doral, FL, USA) in vegetable capsule number 1 with microcrystalline cellulose excipient in the following proportions:

- Placebo—0% Pep19, 74% Excipient, 26% Capsule
- 2 mg—1% Pep19, 73% Excipient, 26% Capsule
- 5 mg—2.5% Pep19, 71.5% Excipient, 26% Capsule

### 2.2 Study Design and Participants

The present study was carried out following approval by the Ethics Committee of the Advarra Institutional Review Board (Pro00075623), and registered online at the National Library of Medicine (NIH; https://clinicaltrials.gov/study/NCT06359327). All subjects provided their written informed consent to participate in the study. A sample size of at least 8 subjects per group was based on a 10% mean difference and 80% power calculation.

For this study, the inclusion criteria were generally healthy males and females between the ages of 40 and 70 years old, and BMI between 30 and 35 kg/m^2^. Participants in this study were not specifically stratified based on BMI before group assignment. However, the BMI data for the participants were closely matched across the groups, with no significant differences in baseline BMI values observed. This ensured that the BMI ranges for each group overlapped sufficiently to facilitate comparison across the study arms.

The exclusion criteria were pregnancy, lactation and the use of anti‐obesity supplements or medications; female menopausal status was not an exclusion criterion. Subjects were selected from the Precision Clinical Research Centre (PCRC; Sunrise, FL, USA) database. Subjects that matched inclusion criteria were contacted to provide information about the study, and if they met the preliminary qualifications based on a phone interview, they were invited to the clinic for a screening visit. At the clinic, their demographic information and medical history were collected, and if they met the eligibility criteria, they proceeded with initial screening. All the studies were conducted from May to August 2024 at PCRC.

Healthy males (*n* = 12) and females (*n* = 12), ages varying from 46 to 59 years, weighing in the range of 91–106 kg, and with BMI between 30 and 35 kg/m^2^, were selected for the study. During screening, all subjects provided complete medical histories, and underwent a physical examination, anthropometric assessment, and routine laboratory tests to determine eligibility and ensure safety; most of these subjects reported poor sleep quality. Individuals were not included if they were using any type of medication for weight loss, or were pregnant, or lactating. As subjects achieved the eligibility criteria, they were sequentially included in the groups. The convenience sampling method was chosen due to logistical constraints; however, this may have introduced selection bias. To mitigate this, future studies should consider random or stratified sampling.

Twenty‐four subjects were assigned to the placebo, 2 mg Pep19, or 5 mg Pep19 groups, with treatments administered orally once daily at bedtime, in a 1:1:1 ratio, for 60 days, in a triple‐blinded (subjects, researchers and data analysis) fashion. No specific BMI stratification was performed before the group selection. Subjects underwent a repeat physical examination and anthropometric assessment on day 0 (baseline) and day 60 (post‐intervention).

Subjects were informed that their identities would not be linked to their responses (anonymity), and that they were free to stop participating in the study at any point without any negative repercussions. Subjects received 30 capsules at the initiation of the study, and another 30 capsules after 30 days during the follow‐up visit to PCRC. All subjects were instructed to self‐administer the received capsules once a day at bed time, in a triple‐blinded fashion.

### 2.3 Assessments and Anthropometric Measurements

All the following procedures were conducted at the PCRC using standard protocols. Compliance was monitored by the number of capsules returned on the 1st‐month and 2nd‐month visits. All measures were taken at baseline and 60 days.

The primary endpoint was to assess the impact of Pep19 in the broad definition of the quality of life of obese subjects, which includes sleep quality. Key secondary endpoints included body composition measurements (body weight, height, lean mass, fat mass, and circumferences). Exploratory secondary endpoints focused on physical (e.g., blood pressure) and biochemical markers related to obesity. These endpoints provided a comprehensive evaluation of Pep19's effects on the multifaceted consequences of obesity.

Primary endpoints, health‐related quality of life and sleep quality, were assessed using validated questionnaires: the Short Form Health Survey (SF‐12) and the Pittsburgh Sleep Quality Index (PSQI). SF‐12, a widely used tool, validated to the USA obese population (http://www.cdc.gov/pcd/issues/2008/apr/07_0051.htm), includes 12 questions covering physical and mental health, yielding two summary scores: the Physical Component Summary (PCS) and Mental Component Summary (MCS) [38]. Scores ranged from 0 to 100, with higher scores indicating better health. Sleep quality was assessed using the Pittsburgh Sleep Quality Index (PSQI), a well‐validated 19‐item questionnaire extensively used across diverse populations, including individuals with obesity, ageing‐related conditions, insomnia, and depression. The PSQI generates seven component scores assessing sleep quality, latency, duration, efficiency, disturbances, medication use, and daytime dysfunction. Each component is scored from 0 to 3, with higher scores indicating poorer sleep quality. The total PSQI score ranges from 0 to 21, with scores > 5 denoting poor sleep quality [39].

The key secondary endpoints were reduction in visceral fat and weight loss, in addition to waist, hip and chest measurements.

Fat mass and lean mass (kg) were assessed using a Dual‐Energy X‐ray Absorptiometry (DXA) scan performed on a Hologic Horizon DXA machine (Horizon‐Wi serial number 302284M, Hologic, Marlborough, MA, USA) [40]. A standard whole‐body scan was conducted with the participant positioned in a supine position. Calibration of the DXA machine was performed daily, following the manufacturer's protocol. Fat mass index (g/cm^2^) was defined as the total DXA fat mass normalised by height squared (total fat mass/height [2]); fat mass index has a distinct advantage over BMI for defining obesity status since it is independent of lean mass status [40].

Weight was measured using a calibrated digital scale. Participants were asked to remove heavy clothing, shoes, and accessories, and to stand still at the centre of the scale while wearing light clothing. These measurements were recorded in kg.

Waist, hip and chest measurements were recorded in cm. Briefly, waist circumferences were measured using a flexible, non‐stretchable tape measure. The measurements were taken at the midpoint between the lowest rib and the iliac crest, ensuring that the tape was snug but not compressing the skin, and parallel to the ground. The participants stood upright with their feet together and arms relaxed at their sides during measurements. Waist circumferences were recorded in cm. Hip circumferences were measured at the widest part of the hips and buttocks, usually at the level of the greater trochanters. The tape was placed snugly, but not tightly, around the hips, ensuring it was parallel to the ground and not compressing the skin. The participants stood upright, feet together, and arms relaxed at their sides. Chest circumferences were measured at the fullest part of the chest, typically around the nipple line or just above the nipples. The flexible tape measure was positioned snugly but without compressing the skin, ensuring it was parallel to the ground for accurate placement.

Exploratory secondary endpoints assessed health‐related obesity parameters and safety. Blood pressure, heart rate and body temperature were measured by clinic staff. Blood pressure was recorded using an automatic digital machine with the cuff placed 2.5 cm above the elbow crease, ensuring proper positioning at the heart level; the device is calibrated annually. Results of blood pressure were presented as systolic blood pressure and diastolic blood pressure (mmHg). Heart rate was recorded simultaneously with the blood pressure measurements. Blood samples were collected after an 8‐h fast using two types of tubes from Quest Diagnostics (Sunrise, FL, USA): an EDTA K2/K3 Tube (4 mL) for immediate inversion and centrifugation at 2000–2500 × g for 10–15 min, and a CAT Serum Sep Clot Activator Tube (8 mL), which was allowed to clot for 30 min before centrifugation. Biochemical analyses were performed by Quest Diagnostics, including glucose (spectrophotometry), insulin (immunoassay), HbA1c (enzymatic assay), alanine aminotransferase (ALT) (spectrophotometry), aspartate aminotransferase (AST) (spectrophotometry), C‐Reactive Protein (immunoturbidimetric), and triglycerides (spectrophotometry). Homoeostasis model assessment of insulin resistance index (HOMA‐IR) was calculated by the formula HOMA‐IR = (glucose [mg/dL] × insulin [μU/L])/22.5 [41].

Below is the schematic design of the study process.


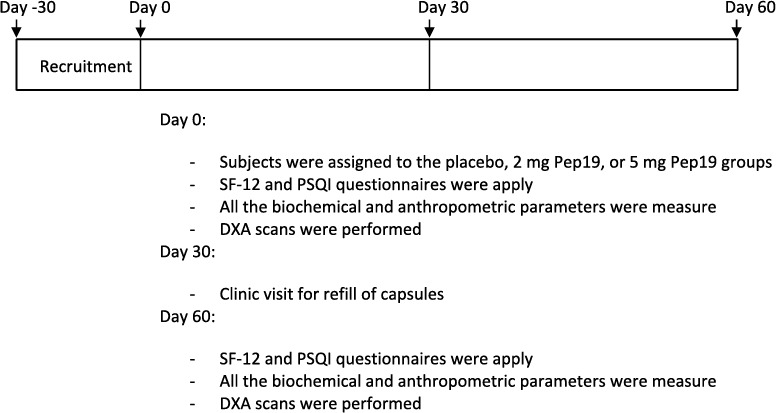


### 2.4 Statistical Analyses

The sample size was chosen using power calculation assuming a mean 10% difference between placebo and 2 or 5 mg groups, a Cohen's *d* = 0.5 with a significance level of *α* = 0.05, and a desired power of 80% using R software [42]. Using this assumption, the study should have at least 8 subjects in each group. The research analyst (AH) remained blinded during the initial analyses of the raw data and received the code only after completing analysis of weight, waist circumference, body composition (i.e., DXA), blood pressure, quality of life, and sleep quality. In addition, two subsets were analysed: (1) for blood pressure, we analysed the subset that was not taking antihypertensive medication; (2) for sleep quality, we analysed the subset that started with a total PSQI score over 5 [39]. Data were described as mean and standard deviation or standard error of the mean (SEM) at baseline (initial measurements before starting taking placebo or Pep19). Follow up data were described as a percentage of baseline; individual measurements at initiation were considered 100%; at 60 days the relative percentage was calculated as the percentage of the baseline/initial value. Data were analysed using one way ANOVA with Tukey's multiple comparison test using GraphPad Prism (GraphPad Software, www.graphpad.com). Significance was set to *p* < 0.05.

## 3 Results

### 3.1 Characteristics of the Present Study Participants

Twenty‐four obese adults with generally normal vital signs, and most of them with poor sleep quality, were enrolled in the study (Table 1). Subjects were blindly instructed to self‐administer either placebo, Pep19 2 mg, or Pep19 5 mg daily dose for the next 60 days at bedtime. One subject from the 2 mg Pep19 sub‐group dropped out for administrative reasons, without any relationship with any reported undesired adverse effect (Table 1). Although the group receiving 5 mg of Pep19 exhibited an imbalance in the number of males and females as well as a seemingly higher initial weight and height, no statistically significant differences among variables were observed between the groups; the sample size did not allow a separate statistical analysis to compare males and females (Table 1). None of the subjects reported any adverse undesired effects during the present study.

**TABLE 1:** Baseline demographic, anthropometric, biochemical and body measurements.

	Placebo (*n* = 8)	2 mg Pep19 (*n* = 7)	5 mg Pep19 (*n* = 8)
Age (years)	55 ± 4	53 ± 5	52 ± 6
Female	5	5	2
Male	3	2	6
Weight (kg)	94 ± 12	91 ± 10	100 ± 6
Height (cm)	169 ± 10	165 ± 7	178 ± 6
Body‐mass index (kg/m^2^)	33 ± 1	33 ± 1	32 ± 1
Visceral fat (kg)	1.04 ± 0.14	1.07 ± 0.19	1.08 ± 0.12
Fat free mass (kg)	47 ± 7	45 ± 11	56 ± 7
Fat index (fat mass/height^2^; g/cm^2^)	1.46 ± 0.22	1.51 ± 0.20	1.20 ± 0.22
Waist circumference (cm)	107 ± 8	109 ± 6	111 ± 5
Hip circumference (cm)	117 ± 6	115 ± 6	112 ± 7
Chest circumference (cm)	113 ± 9	114 ± 6	115 ± 5
Temperature (°C)	37 ± 0.2	37 ± 0.3	36 ± 0.2
Heart rate (beats per minute)	76 ± 10	69 ± 9	72 ± 12
Systolic blood pressure (mmHg)	122 ± 12	128 ± 6	127 ± 5
Diastolic blood pressure (mmHg)	79 ± 9	84 ± 6	81 ± 7
Sleep (Pittsburgh sleep quality index)	7 ± 3	9 ± 3	8 ± 2
SF‐12 mental component score	49 ± 6	44 ± 13	42 ± 8
SF‐12 physical component score	54 ± 10	46 ± 12	48 ± 6
Glucose (mg/dL)	103 ± 30	99 ± 18	96 ± 10
Insulin (μU/mL)	21 ± 27	14 ± 9	17 ± 7
HOMA‐IR	130 ± 227	65 ± 52	72 ± 34

*Note:* No statistically significant differences (*p* < 0.05) were observed among the groups at baseline. Data are mean ± standard deviation; *n* = 7–8.

Abbreviation: HOMA‐IR, Homoeostasis model assessment of insulin resistance index.

### 3.2 Biochemical Parameters

The biochemical analyses showed a large variation among subjects, and no statistically significant changes could be observed among these groups (Table 2). Although it was not statistically significant, increased ALT and AST plasma levels in the male group receiving 5 mg of Pep19 need further investigation, as it may be an indication of a possible adverse effect of Pep19 (Table 2).

**TABLE 2:** Biochemicals measured at initial baseline (0) and after 60 days.

	Placebo (*n* = 8)		2 mg Pep19 (*n* = 7)		5 mg Pep19 (*n* = 8)	
Time (days)	0	60	0	60	0	60
Triglycerides (mg/dL)	184 ± 130	136 ± 90	127 ± 32	137 ± 48	184 ± 101	189 ± 114
ALT (Female) (U/L)	19 ± 3	20 ± 5	19 ± 10	20 ± 13	18 ± 0	16 ± 0
AST(Female) (U/L)	18 ± 3	19 ± 3	18 ± 5	20 ± 7	16 ± 0	18 ± 0
ALT (Male) (U/L)	16 ± 5	13 ± 3	22 ± 2	23 ± 1	29 ± 12	38 ± 42
AST (Male) (U/L)	13 ± 2	12 ± 0	18 ± 2	19 ± 1	20 ± 6	32 ± 31
C‐reactive protein (mg/L)	5 ± 3	8 ± 2	4 ± 1	5 ± 1	3 ± 0	7 ± 3
Glucose (mg/dL)	103 ± 30	99 ± 14	99 ± 18	107 ± 27	96 ± 10	108 ± 24
Insulin (μU/mL)	21 ± 27	8 ± 3	14 ± 9	12 ± 7	17 ± 7	12 ± 5
HgBA1c (%)	6 ± 0	6 ± 1	6 ± 0	6 ± 1	6 ± 0	6 ± 0

*Note:* No statistically significant differences (*p* < 0.05) were observed among the groups. Data are mean ± standard deviation, *n* = 7–8.

Abbreviations: ALT, Alanine aminotransferase; AST, aspartate aminotransferase.

### 3.3 Health‐Related Quality of Life and Sleep Quality

**FIGURE 1:**
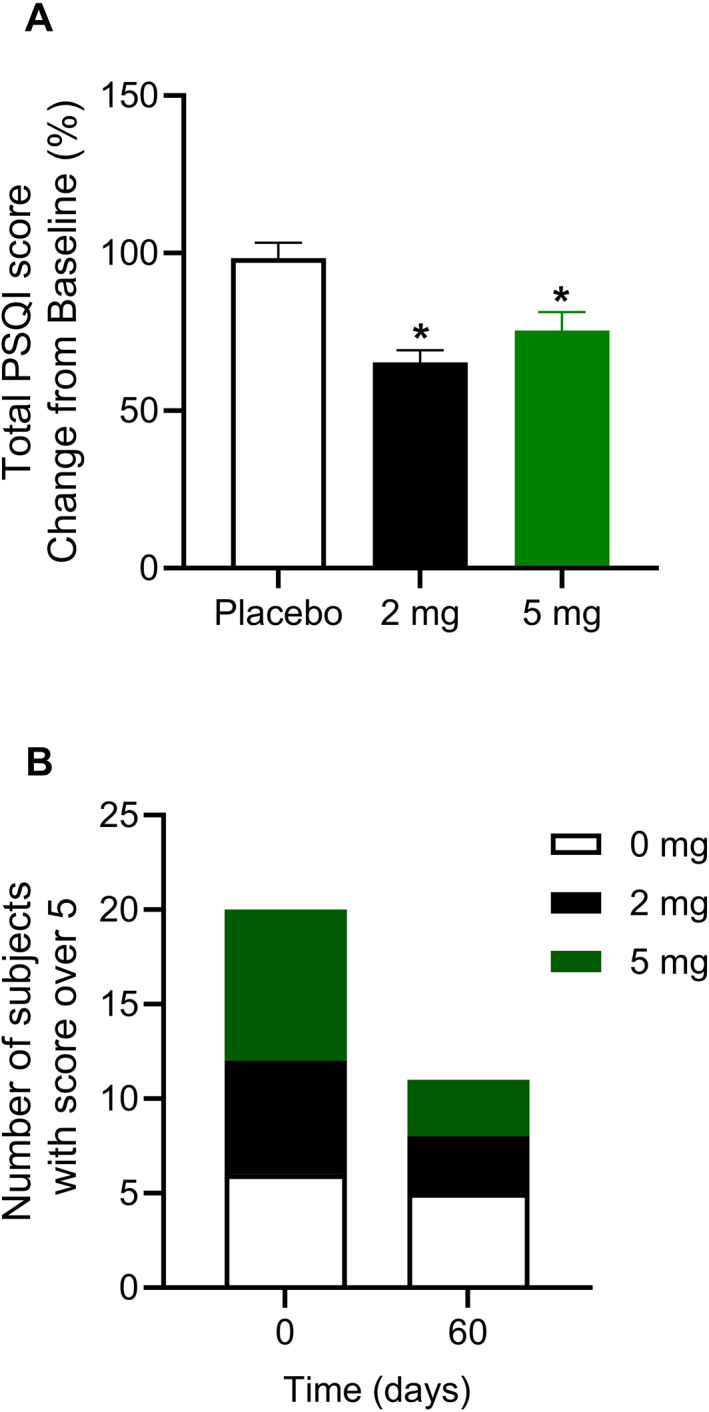
Sleep quality over time. Panel A: changes in total score at day 60 (mean ± standard error of the mean [SEM]); *Pep19 versus placebo; *p* < 0.05. Note that subjects decreased global PSQI score (i.e., have improved sleep quality) in 35% (SEM = 10%) and 25% (SEM = 16%), respectively, receiving either 2 mg Pep19 or 5 mg Pep19 capsules. Panel B: number of subjects with PSQI score over 5. Subjects were assigned to receive either placebo (Placebo), 2 mg Pep19 (2 mg), or 5 mg Pep19 (5 mg) self‐administered daily at bedtime, for 60 days, in a triple‐blinded fashion. Pep19 and placebo capsules were both formulated by Nutroslim LLC (Doral, FL, USA) as a dietary supplement, in vegetable capsules with microcrystalline cellulose excipient. Data are mean ± SEM; *n* = 7–8.

No changes were observed in the health‐related quality of life of the subjects as assessed with the SF‐12, one of the primary endpoints. As for the other primary endpoints linked to the quality of life of obese subjects, groups receiving either 2 mg Pep19 or 5 mg Pep19 capsules decreased global PSQI score (i.e., have improved sleep quality), respectively, in 35% (SEM = 10%) and 25% (SEM = 16%) (Figure 1A).

We assessed sleep quality among all study participants. Initially, 6 of 8 subjects in the placebo group, 6 of 7 in the 2 mg Pep19 group, and 8 of 8 in the 5 mg Pep19 group reported poor sleep quality. After 60 days, from the subjects that initially reported poor sleep, 1 of 6 participants in the placebo group improved sleep (Figure 1B). However, 3 of 6 in the 2 mg Pep19 group, and 5 of 8 in the 5 mg Pep19 group no longer exhibited poor sleep quality (Figure 1B). These data represent a 43% and 63% reduction in subjects with poor sleep for those taking 2 mg Pep19 and 5 mg Pep19 capsules, respectively, compared to a 13% reduction with placebo treatment.

### 3.4 Body Composition, Anthropometric and Weight Measures

**FIGURE 2:**
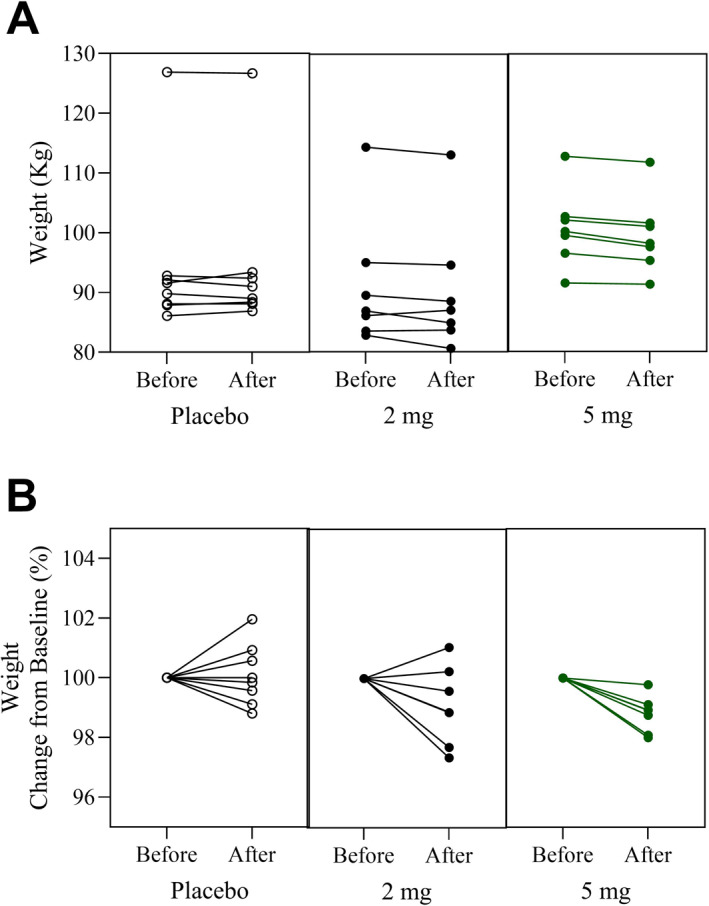
Body weight on day 0 (before) and on day 60 (after) relative to baseline. Panel A: body weight (kg) of each subject before and after administering placebo (Placebo), 2 mg Pep19 (2 mg), or 5 mg Pep19 (5 mg), self‐administered daily at bedtime, for 60 days, in a triple‐blinded fashion. Panel B: body weight relative to baseline (change from baseline %) for each subject. Subjects received either placebo (Placebo), 2 mg Pep19 (2 mg), or 5 mg Pep19 (5 mg) self‐administered daily at bedtime, for 60 days, in a triple‐blinded fashion. Pep19 and placebo capsules were both formulated by Nutroslim LLC (Doral, FL, USA) as a dietary supplement, in vegetable capsules with microcrystalline cellulose excipient.

A reduction in body weight and waist circumference (*p* < 0.05) was observed in subjects assigned to 5 mg Pep19 compared to both baseline measurements and placebo (Table 3 and Figure 2). A slight reduction in chest circumference and fat index was also observed for the 5 mg Pep19 group compared with the placebo group (*p* = 0.07; Table 3).

**TABLE 3:** Body measurements after 60 days relative to initial baseline measurements (%).

	Placebo (*n* = 8)	2 mg Pep19 (*n* = 7)	5 mg Pep19 (*n* = 8)
Body weight	100 ± 1	99 ± 1	99 ± 0.6^a^ ^,^ ^b^
Waist circumference	100 ± 1	99 ± 2	98±1^a^ ^,^ ^b^
Chest circumference	100 ± 1	99 ± 2	99±1^c^
Hip circumference	100 ± 1	99 ± 1	99 ± 1
Fat mass index	103 ± 3	103 ± 5	101±1^c^
Heart rate	99 ± 17	100 ± 9	103 ± 8
Systolic blood pressure	102 ± 7	101 ± 4	97 ± 6
Diastolic blood pressure	106 ± 15	102 ± 4	99 ± 12
Visceral fat	102 ± 5	105 ± 6	83±5^a^ ^,^ ^b^
Fat free mass	97 ± 4	98 ± 5	97 ± 3

*Note:* Data are mean ± standard deviation, *n* = 7–8.

^a^ *p* < 0.05 versus baseline.

^b^ *p* < 0.05 versus placebo.

^c^ *p* = 0.07 versus placebo.

**FIGURE 3:**
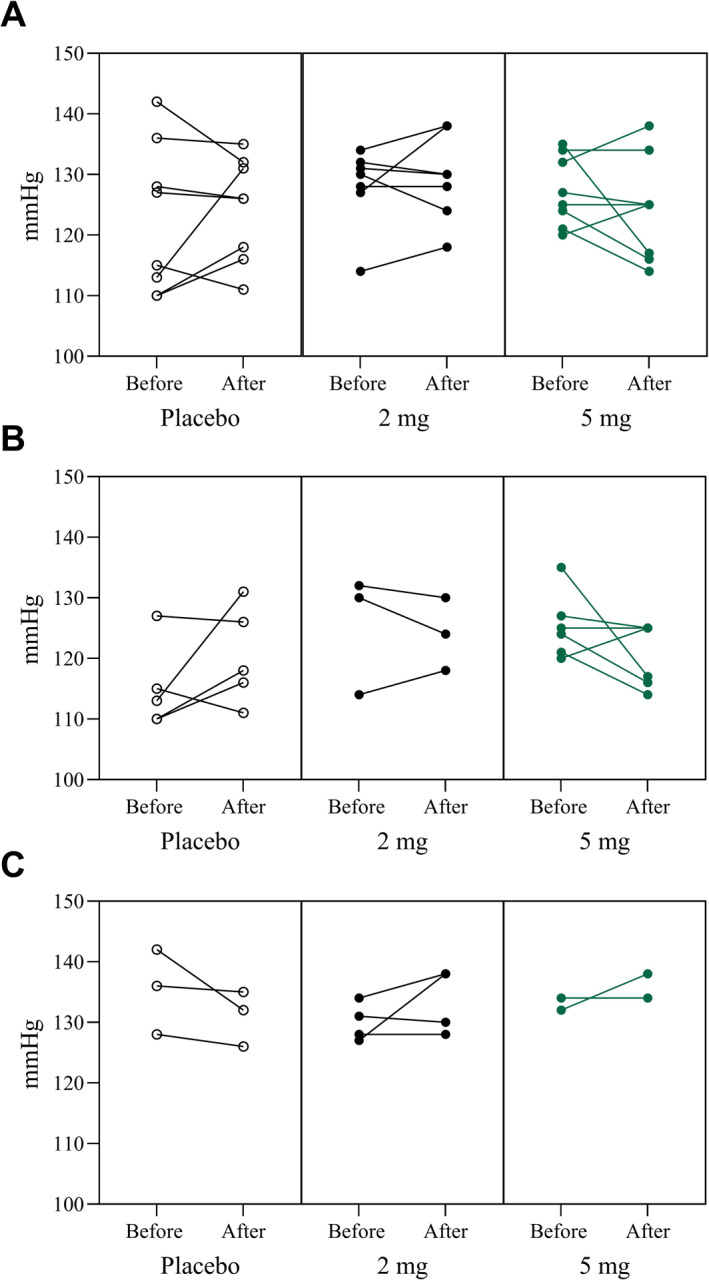
Systolic blood pressure (mmHg) over time. Subjects received either placebo (Placebo), 2 mg Pep19 (2 mg), or 5 mg Pep19 (5 mg) self‐administered daily at bedtime, for 60 days, in a triple‐blinded fashion. Panel A: changes in systolic blood pressure (SBP) of each subject of the study. Panel B: changes in SBP from subjects that were not taking antihypertensive drugs. Panel C: changes in SBP from subjects that were taking antihypertensive drugs.

Heart rate, systolic blood pressure, and diastolic blood pressure were measured in all participants. Overall, there were no statistically significant changes in blood pressure or heart rate after 60 days of Pep19 when compared to baseline measurements and the placebo group (Table 3 and Figure 3). However, in the subset of subjects not taking antihypertensive medication (5 of 8 in the placebo group, 3 of 7 in the 2 mg Pep19 group, and 6 of 8 in the 5 mg Pep19 group) after 60 days, there was a notable trend towards decreased systolic blood pressure in the 5 mg Pep19 group compared to placebo (*p* = 0.07), and also compared to baseline (*p* = 0.08) (Table 4 and Figure 3).

**TABLE 4:** Blood pressure from a subset of subjects that were not on antihypertensives.

	Placebo (*n* = 5)	2 mg Pep19 (*n* = 3)	5 mg Pep19 (*n* = 6)
Systolic blood pressure (% of baseline)	105 ± 8	99 ± 4	96 ± 7 (*p* = 0.07)
Diastolic blood pressure (% of baseline)	110 ± 18	102 ± 4	99 ± 13

*Note:* Data relative to baseline measurements (%). No statistically significant differences (*p* < 0.05) were observed among the groups. Table 1 shows the raw data of blood pressure measurements. Data are mean ± standard deviation; *n* = 3–6.

### 3.5 Visceral Fat and Lean Mass Assessments

**FIGURE 4:**
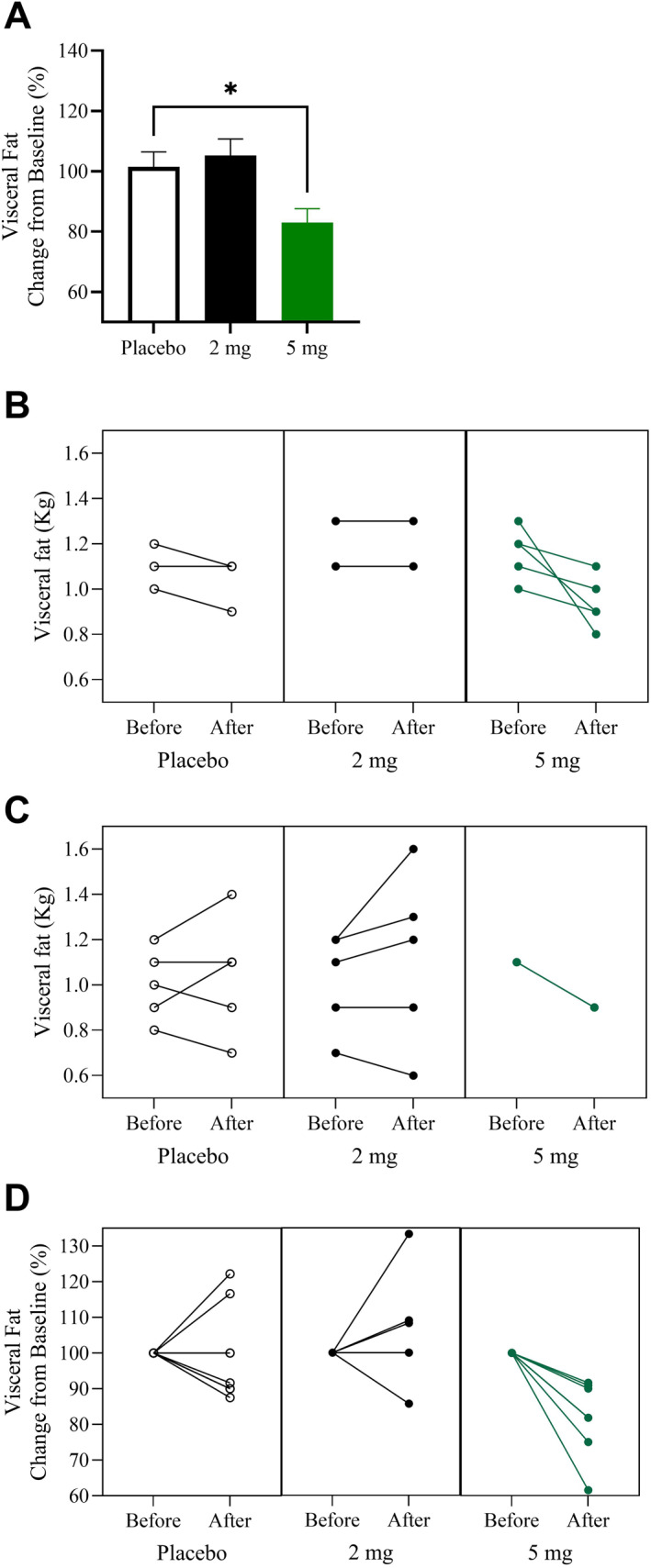
Body composition was measured using DXA on day 0 (before) and on day 60 (after). Panel A: visceral fat change from baseline (mean ± standard error of the mean [SEM]) (*p < 0.05, compared to placebo). Panel B: visceral fat of each male subject (kg); Panel C: visceral fat of each female subject (kg). Panel D: visceral fat change from baseline of each subject (%). Subjects received either placebo (Placebo), 2 mg Pep19 (2 mg), or 5 mg Pep19 (5 mg) self‐administered daily at bedtime, for 60 days, in a triple‐blinded fashion. Pep19 and placebo capsules were both formulated by Nutroslim LLC (Doral, FL, USA) as a dietary supplements in vegetable capsules with microcrystalline cellulose excipient. Note that in the 5 mg group, all subjects lost visceral fat after 60 days of Pep19. Data are mean ± SEM; *n* = 7–8.

Visceral fat and lean mass composition were assessed using a DXA scan. A major reduction in visceral fat mass was observed in the 5 mg Pep19 group, measured at 60 days and compared to both baseline and placebo measurements (Table 3, Figure 4). No changes in lean mass were observed with either 2 mg Pep19 or 5 mg Pep19 administration compared with baseline and placebo measurements (Table 3). Note that all the subjects on the 5 mg Pep19 group experienced a decrease in visceral fat, which was not observed on groups assigned to placebo or 2 mg Pep19 (Figure 4).

## 4 Discussion

A Pep19‐based therapeutic strategy holds significant innovative potential as a breakthrough in visceral fat and sleep quality management, offering several unique advantages. Remarkably, Pep19 shows important advantages over similar compounds, including the ability to reduce visceral fat without altering the lean mass, and the absence of undesired adverse effects. Given that visceral fat contributes to determinants in cardiometabolic health across BMI categories, Pep19 may be recommended to improve health not only in obese subjects but also in individuals normal and overweight with high amounts of visceral fat as well. Pep19 effects were largely mediated by a reduction in fat mass, whereas there was no detectable difference in lean mass. Considering the importance of maintaining lean mass during weight loss, Pep19 may be safely taken in all age groups including elderly. Considering the impact of Pep19 on sleep, individuals with poor sleep may benefit from its consumption; importantly, poor sleep remains highly prevalent and pervasive in modern society. In accordance with established scientific protocols and FDA guidelines, Pep19 is considered safe for human consumption. Both the current and previous spontaneous reporting analyses found no evidence of adverse effects. This study further advances our understanding of Pep19's therapeutic potential and safety profile.

Visceral fat accumulation has emerged as a significant public health concern, primarily due to its association with increased risks for metabolic disorders such as type 2 diabetes, cardiovascular disease, and non‐alcoholic fatty liver disease [23, 24, 25, 29]. Epidemiological studies indicate that approximately 30% of the global adult population is affected by obesity, with a substantial proportion exhibiting increased visceral adiposity [43]. High visceral fat increases long‐term mortality and low‐grade inflammation, and helps identify individuals most at risk of long‐term mortality due to visceral inflammatory obesity [25]. Notably, visceral fat is more prevalent in individuals with abdominal obesity, particularly those over 40 years of age, showing a higher tendency to accumulate fat in the abdominal region [43]. Given the significant role that visceral fat plays in the pathogenesis of these chronic diseases, there is a pressing need for novel therapeutic approaches targeting its reduction. Current treatment strategies, including lifestyle interventions, bariatric surgery, and GLP‐1‐based therapies, often prove ineffective over the long term for a significant portion of the population. This underscores the urgent need for novel therapeutic options to address this growing clinical challenge. Given that obesity is a chronic, physiologically regulated condition, sustained weight loss is essential for achieving long‐term health. Therefore, identifying and developing targeted therapies aimed at reducing visceral fat could have a profound impact on alleviating the global burden of metabolic diseases and improving the overall public health.

Here, capsules containing 5 mg Pep19, self‐administered by subjects at bedtime for 60 days, were associated with a substantial loss of visceral fat (17% in average) without changing the lean mass. The Pep19 5 mg group consistently exhibited weight loss compared to the placebo group. These exciting findings of Pep19 reducing visceral adipose tissue and body weight in human subjects, without affecting the lean mass, mirror Pep19 effects previously observed and molecularly characterised in adipose cells and experimental animal models [17, 22, 35]. Previous evaluations of Pep19 in animal models demonstrated no effects on behaviour, locomotor activity, or energy intake, following both acute and chronic administration [17, 22, 35]. Considering that Pep19 effects observed in animal models were successfully translated to humans in the present study, it is highly unlikely that the observed changes in body composition or weight can be attributed to alterations in behaviour (e.g., reduced physical activity) or energy intake in the investigated subjects. Altogether, these findings reinforce that the observed changes reported herein are specifically attributable to the Pep19 intervention, rather than to external factors such as increased physical activity or reduced energy intake. However, further studies are needed to confirm these findings and better understand the long‐term effects and underlying Pep19 mechanism of action.

The mechanism of action of Pep19 was initially proposed through biochemical analysis using conformationally sensitive antibody screening, and subsequently confirmed in cell culture assays and animal models [17, 22, 35]. Pep19 is an intracellular peptide that activates UCP1 [22]. Several distinctive intracellular peptides derived from either TSC22 domain family protein 1, bromodomain and WD repeat‐containing protein 1, protein piccolo, or collagen alpha‐1 (III) chain, identified in human fetal interscapular brown adipose tissue primary pre‐adipocytes, were suggested to be associated with increased UCP1 gene expression [44]. Although the extent of the browning thermogenic effect is different between rodent models compared to humans, due to the difference in adipose tissue distribution, this mechanism of action has been previously shown to be conserved among species [45, 46]. White adipose tissue browning induces fat loss and remodelling through an increased activation of UCP1 gene expression [22, 47], which could be one of the possible Pep19 mechanisms of action in humans, as previously shown to occur in cellular and animal models [17, 22]. The selective reduction of visceral fat induced by Pep19 in obese subjects represents a mechanistically distinct approach to weight management, particularly for individuals unable to tolerate conventional therapies, such as glucagon‐like peptide‐1 (GLP‐1) receptor agonists (e.g., semaglutide and tirzepatide), which are associated with significant lean mass loss [48, 49]. However, a key limitation of this study is its relatively small sample size, which may constrain the detection of rare or subtle adverse effects, including potential impacts on lean mass. While the primary findings provide valuable insights, studies with larger cohorts and extended follow‐up periods are warranted to further characterise the safety profile of Pep19 and improve the generalisability of these results.

In the context of metabolic regulation and body composition in obese adults investigated herein, it is important to consider the role of GLP‐1 receptor agonists, such as semaglutide and tirzepatide [50, 51]. These drugs have emerged as highly effective therapies for obesity and metabolic disorders, demonstrating significant weight‐loss benefits through mechanisms involving appetite suppression, delayed gastric emptying, and improved glycaemic control [52]. Further investigation is required to elucidate the potential cross‐interaction between Pep19 and GLP‐1 receptor agonists, such as semaglutide and tirzepatide, and their influence on metabolic and neuroendocrine processes at the molecular level. The amino acid sequence of Pep19 (DIIADDEPLT) minimises the potential for competitive inhibition of dipeptidyl peptidase IV (DP IV, CD26, EC 3.4.14.5), thereby reducing indirect effects associated with increased circulating GLP‐1 levels; DP IV cleaves substrates containing X‐Pro or X‐Ala at the N‐terminus [53]. Pep19 has not yet been tested for endocrine GLP‐1‐like activity or agonist action on GLP‐1 receptors. Thus, further investigations could provide valuable insights into the possibly broader physiological effects and therapeutic applications of Pep19.

Previous studies have demonstrated that certain bioactive peptides retain their biological activity following oral administration, including hemopressins [15, 54, 55], NFKF [56, 57], haemoglobin alpha subunit derived peptide Ric4 [58], peptides C111 and C112 from bonito liver [59], as well as IPP, VPP, and tryptic peptides derived from casein [60, 61]. It is plausible that a similar mechanism underlies the preservation of Pep19 oral activity, although the specific pathways involved remain largely unknown. Pep19, hemopressin, NFKF, and Ric4 were originally identified as proteasome‐derived intracellular peptides, which may have evolved specific structural features to facilitate efficient absorption while also resisting longer to further proteolytic degradation [6, 9, 12, 18, 62].

Overall, Cohen's calculations were instrumental in determining the appropriate sample size to ensure that the present study was adequately powered to detect clinically relevant effects, while also highlighting the limitations of the sample size for certain exploratory outcomes. Particularly, the higher variability in biochemical analyses reduced the ability to draw definitive conclusions from them. For example, an increment in ALT and AST average levels at the 5 mg dose of Pep19 in male subjects was observed. However, prior rodent studies demonstrated Pep19's potential to improve liver function [17], suggesting that these observed increases in ALT and AST could be minor, though it strongly suggests a need for closer monitoring of liver function and composition in future trials.

The relationship between visceral fat accumulation and sleep quality is complex and bidirectionally related to metabolic health [30]. Notably, sleep deprivation and short sleep duration were implicated in increased visceral and abdominal fat [63, 64]. On the other hand, visceral fat accumulation contributes to chronic inflammation and insulin resistance, factors that also impair sleep quality. Semaglutide and tirzepatide have been reported to improve sleep in some overweight people, which may be related to the reduction in body mass or to direct effects of these drugs controlling airway muscle tone or upper airway patency [65, 66]. Therefore, mitigating visceral fat accumulation could be an effective strategy to improve sleep quality, and conversely, improved sleep quality may help reduce visceral fat, leading to overall improvements in metabolic health. Pep19 exerts its effects on adipocytes by modulating CB1R receptors, which activate UCP1 and drive subsequent fat remodelling. While reductions in adipose tissue content are measurable only after extended treatment, physiological changes such as decreased chronic inflammation and improved insulin sensitivity likely occur earlier. Supporting this, a reduction in insulin resistance has been observed in animal models treated with Pep19, which may also contribute to the improvement in sleep quality [17, 22]. The direct effects of Pep19 on breathing and airway muscle tone remain uninvestigated. Further research is needed to elucidate the mechanisms underlying these effects and to determine whether reductions in chronic inflammation and insulin resistance mediate the observed outcomes of Pep19.

In the present study, administration of either 2 mg Pep19 or 5 mg Pep19 improved sleep quality. 5 mg Pep19 was more effective in restoring sleep quality to the normal range, while also demonstrating a statistically significant reduction in visceral fat and body weight. The 2 mg dose, though not statistically significant (*p* > 0.05), showed a consistent trend towards body weight reduction. The absence of undesired adverse effects following both acute and chronic administration of Pep19 was observed in multiple animal models [17, 22, 35]. Furthermore, several individuals who were taking 5 mg Pep19 in the form of supplement capsules once a day at bedtime independently and spontaneously reported weight and waist circumference loss along with improved sleep quality, without any adverse undesired effects related to Pep19 administration. These findings suggest that the enhancement of sleep quality induced by Pep19 may primarily result from its peripheral effects, including the reduction of visceral adipose tissue and body weight, rather than a direct action on the central nervous system. However, the current data do not allow for a definitive correlation between visceral fat reduction and improved sleep quality. These findings underscore the critical need for integrated approaches that consider the improvement of both sleep and metabolic health in the treatment and prevention of obesity. It thus provides an important new therapeutic strategy to reduce visceral fat and to improve sleep in obese individuals with poor sleep quality. Further clinical trials are required to better understand the extent of Pep19's impact on sleep quality and the interconnection with decreased visceral fat.

## 5 Strengths and Limitations of the Present Study

Our present study has several strengths. It was the first early‐stage clinical trial, placebo‐controlled, triple‐blinded, evaluating two doses of Pep19 (2 and 5 mg) administered at bedtime over 60 days. We used DXA imaging to assess changes in body composition and fat distribution, providing robust, objective measurements. These strengths contribute to the validity of the primary and key secondary endpoints. The unique oral bioavailability of Pep19, taken once a day at bedtime, offers promising potential for future therapeutic applications of intracellular peptides.

While the effects of Pep19 administration on total body weight were modest, the remarkable 17% reduction in visceral fat is a significant finding, given the strong link between visceral fat and cardiometabolic risk, immunity, and mortality [29]. These results suggest that Pep19 may have a broad impact on public health, particularly as part of a therapeutic strategy for obesity and its associated complications.

However, the present study also has notable limitations. The relatively small sample size limits the generalisability of the findings, particularly to diverse populations in terms of geographic and demographic characteristics. The sample size for this study was determined using power calculations, a critical aspect in experimental design to ensure sufficient statistical power to detect meaningful differences between groups. Power calculations are based on a set of assumptions regarding the expected effect size, the level of significance (*α*), and the desired power of the test. In this case, we assumed a 10% mean difference between the placebo and treatment groups, which was considered a clinically relevant effect. Cohen's *d* of 0.5 was chosen, representing a medium effect size according to Cohen's conventions, which is commonly used as a benchmark for determining the sample size in clinical studies. The calculations were conducted with an α level of 0.05, which indicates a 5% risk of type I error (i.e., falsely rejecting the null hypothesis), and 80% power, meaning there was an 80% probability of detecting a true effect if one existed. These assumptions were grounded on unpublished 30‐day open‐label trial data, which were extrapolated to predict the potential outcomes over a 60‐day period. While the sample size calculated based on these parameters was adequate to assess the primary and key secondary endpoints, it may have been insufficient for exploratory analyses, particularly those involving biochemical measures.

There was an imbalance in the 5 mg Pep19 group in terms of gender (6 males vs. 2 females), weight (on average heavier), and height (on average taller), which could have influenced the results. Also, the relatively short 60‐day study period does not provide insights into the long‐term effects of Pep19. Longer administration to a larger number of subjects could potentially lead to more pronounced effects on body weight, waist circumference, blood pressure, and serum markers. Therefore, extended clinical trials are needed to assess the sustained benefits of Pep19; indeed, a 90‐day clinical trial with a larger number of subjects per group is currently underway.

Another significant limitation is the absence of data on physical activity and energy expenditure, caloric intake and dietary habits to better isolate the effects of Pep19 on weight and body composition. These limitations restrict our ability to draw firm conclusions regarding the mechanisms underlying the observed effects. While previous studies on animal models [17, 22, 35] strongly suggest that changes observed herein were likely due to Pep19, we cannot exclude the possibility that the placebo group may have altered their exercise levels or caloric intake, which could have influenced the results. Without these data, the potential role of thermogenesis in visceral fat reduction remains speculative.

An additional key limitation of this study was the homogeneity of the sample, as participants were exclusively residents from Florida, USA. This restricts the generalisability of these findings to broader populations. Additionally, the present study relies on self‐reported data, which may introduce biases such as recall errors or social desirability effects. Future research on Pep19 will aim to include more diverse populations in multicentric clinical trials, and incorporate objective measures to enhance the reliability and applicability of the results.

Future research with an increased sample size should employ regression models to assess the independent effects of visceral fat and sleep quality, accounting for variables such as eating behaviour, race, sex and socioeconomic status.

Further investigations should also explore additional mechanisms by which Pep19 may impact metabolism and overall health. Specifically, the effects of Pep19 on fat distribution, muscle mass, and hormonal regulation warrant additional exploration.

## 6 Conclusions

Pep19 demonstrates substantial potential as a novel therapeutic tool for improving metabolic health, particularly through its targeted reduction of visceral fat, a critical factor in weight management. Beyond its impact on visceral fat reduction, Pep19 also enhances sleep quality, positioning it as a multifaceted intervention for individuals facing obesity, metabolic disorders, and sleep disturbances. These dual benefits set Pep19 apart from conventional weight‐loss therapies, offering a more comprehensive approach to improving overall health.

The simplicity of Pep19's administration (a single capsule at bedtime) together with the absence of reported adverse effects underscores its potential as a highly effective and innovative therapeutic intervention. As a targeted strategy for reducing visceral fat, Pep19 presents a promising alternative to traditional weight‐loss therapies, which focus primarily on overall body weight reduction. Its ability to address obesity‐related complications, particularly those associated with visceral fat and sleep disturbances, positions Pep19 as a unique candidate for improving long‐term metabolic health.

While this study provides compelling preliminary evidence of Pep19's effectiveness, further investigation is necessary to elucidate its mechanisms of action and long‐term clinical efficacy. Future studies should focus on identifying optimal subject populations and refining the therapeutic applications of Pep19. Given the promising findings in both animal models and human subjects, Pep19 could represent a clinical breakthrough in addressing visceral fat reduction and improving sleep quality, two key drivers of obesity‐related morbidity.

Despite some limitations, including the relatively small sample size, the present study strongly supports the potential of Pep19 to improve metabolic health outcomes. This innovative intracellular peptide with oral bioactivity has the potential to become a novel intervention for obesity‐related metabolic and cardiovascular diseases with significant therapeutic implications.

In summary, Pep19 is a promising, safe, and unique intervention that significantly reduces visceral fat, body weight, and waist circumference while improving sleep quality. Its translational effects across cell culture, animal models, and human studies provide a robust foundation for future research. As exploration of Pep19's potential continues, it may prove to be a powerful tool in improving long‐term health outcomes and reshaping the future of metabolic disease management.

## Author Contributions

P.S., A.S.H., A.K. and F.G., designed the study, analysed the data, assisted in the experimental design, executed the experiments, analysed the data, and helped in the interpretation of the results. P.S., A.S.H., E.S.F., A.K. and F.G. discussed the results, commented, and participated in manuscript writing, reviewing and submission.

## Conflicts of Interest

A.S.H., E.S.F. and A.K. are co‐founders of Proteimax Biotechnology Israel Ltd. F.G. is an advisor at Lumen Biosciences, Pep19 Inc. Alt Immuned Inc. DexCom Inc. Slim Health Nutrition, RejuvenateBio Inc. Energesis, Basic Research, Uplifting Results Inc. NovMeta Pharma, Biohaven Pharmaceuticals Inc. GATC Health Corporation, Well Cell Global, Verdant Health. Holds stock or stock options at Slim Health Nutrition, RejuvenateBio Inc. NovMeta, GATC Health Corporation, Verdant Health, and Ketogenic Health Systems. Patents licenced, Slim Health Nutrition. P.S. declares no potential conflicts of interest.

### Peer Review

The peer review history for this article is available at https://www.webofscience.com/api/gateway/wos/peer-review/10.1002/dmrr.70056.

## Data Availability

The data that support the findings of this study are available on request from the corresponding author. The data are not publicly available due to privacy or ethical restrictions.
